# Profiling the circulating miRNAs in mice exposed to gram-positive and gram-negative bacteria by Illumina small RNA deep sequencing

**DOI:** 10.1186/s12929-014-0106-y

**Published:** 2015-01-07

**Authors:** Cheng-Shyuan Rau, Shao-Chun Wu, Johnson Chia-Shen Yang, Tsu-Hsiang Lu, Yi-Chan Wu, Yi-Chun Chen, Siou-Ling Tzeng, Chia-Jung Wu, Ching-Hua Hsieh

**Affiliations:** Department of Neurosurgery, Kaohsiung Chang Gung Memorial Hospital and Chang Gung University College of Medicine, Kaohsiung City, 833 Taiwan; Department of Anesthesiology, Kaohsiung Chang Gung Memorial Hospital and Chang Gung University College of Medicine, Kaohsiung City, 833 Taiwan; Department of Plastic and Reconstructive Surgery, Kaohsiung Chang Gung Memorial Hospital and Chang Gung University College of Medicine, No.123, Ta-Pei Road, Niao-Song District Kaohsiung City, 833 Taiwan

**Keywords:** microRNAs (miRNAs), Circulating microRNAs, Gram-positive bacteria, Gram-negative bacteria, Small RNA deep sequencing

## Abstract

**Background:**

We profiled the expression of circulating microRNAs (miRNAs) in mice using Illumina small RNA deep sequencing in order to identify the miRNAs that may potentially be used as biomarkers to distinguish between gram-negative and gram-positive bacterial infections.

**Results:**

Recombinant-specific gram-negative pathogen *Escherichia coli* (Xen14) and gram-positive pathogen *Staphylococcus aureus* (Xen29) were used to induce bacterial infection in mice at a concentration of 1 × 10^8^ bacteria/100 μL of phosphate buffered saline (PBS). Small RNA libraries generated from the serum of mice after exposure to PBS, Xen14, Xen29, and Xen14 + Xen29 via the routes of subcutaneous injection (I), cut wound (C), or under grafted skin (S) were analyzed using an Illumina HiSeq2000 Sequencer. Following exposure to gram-negative bacteria alone, no differentially expressed miRNA was found in the injection, cut, or skin graft models. Exposure to mixed bacteria induced a similar expression pattern of the circulating miRNAs to that induced by gram-positive bacterial infection. Upon gram-positive bacterial infection, 9 miRNAs (mir-193b-3p, mir-133a-1-3p, mir-133a-2-3p, mir-133a-1-5p, mir-133b-3p, mir-434-3p, mir-127-3p, mir-676-3p, mir-215-5p) showed upregulation greater than 4-fold with a *p*-value < 0.01. Among them, mir-193b-3p, mir-133a-1-3p, and mir-133a-2-3p presented the most common miRNA targets expressed in the mice exposed to gram-positive bacterial infection.

**Conclusions:**

This study identified mir-193b-3p, mir-133a-1-3p, and mir-133a-2-3p as potential circulating miRNAs for gram-positive bacterial infections.

**Electronic supplementary material:**

The online version of this article (doi:10.1186/s12929-014-0106-y) contains supplementary material, which is available to authorized users.

## Background

MicroRNAs (miRNAs) are small regulatory RNA molecules that are approximately 22 nucleotides long, modulate the activity of specific mRNA targets, and play important roles in a wide range of physiologic and pathologic processes [1]. Differential expression of miRNAs may help distinguish between disease states [2-4]. miRNAs are themselves active moieties and should therefore reflect physiological alterations directly, which makes them ideal biomarkers for distinguishing between diseased and healthy subjects [5]. Moreover, circulating miRNAs in the blood are remarkably stable [6] and biochemical analyses indicate that miRNAs are resistant to ribonuclease (RNase) activity, extreme pH and temperature, extended storage, and large numbers of freeze-thaw cycles [7,8]. Circulating miRNAs, which can be easily detected by non-invasive methods, have proven to be potentially valuable biomarkers for a variety of diseases [9,10].

Early diagnosis of potential bacterial infection has become the key approach to dealing with the infection illness and to timely correction of the associated complications. Microbiological culture is the gold standard in distinguishing sepsis from other non-infectious diseases, but this technique is always time-consuming and can delay treatment. Circulating miRNAs have been reported to be associated with sepsis [11,12] and various infection diseases, such as viral exposure [13,14], tuberculosis [15], and parasites [16,17]. A distinct circulating miRNA expression was also found in mice exposed to lipopolysaccharide (LPS) originating in the wall of gram-negative bacteria, as well as to lipoteichoic acid (LTA), a major component of gram-positive bacterial walls [18,19]. In addition, increasing evidence has suggested that circulating miRNAs also have important biologic functions [20]. Recently, the Illumina deep sequencing platform has been used for efficient miRNA discovery, and it is widely used to generate small RNA profiles in various organisms. In this study, we profiled the expression of the circulating miRNAs in a mouse model of gram-negative and/or gram-positive bacterial infection using Illumina small RNA deep sequencing.

## Methods

### Experimental design

Male C57BL/6 mice (age, 10–12 weeks; weight, 30–35 g) were purchased from BioLasco (Yi-Lan, Taiwan). The mice were anesthetized by intraperitoneal injection of an anesthetic cocktail consisting of 0.1 mg/g ketamine and 0.01 mg/g xylazine. The anesthetized mice were restrained in a supine position on a heated pad to maintain body temperature at 37°C. Recombinant-specific gram-negative pathogen *Escherichia coli* (Xen14) and gram-positive pathogen *Staphylococcus aureus* (Xen29) purchased from Caliper (Caliper, USA) were used to induce bacterial infection in the mice at a concentration of 1 × 10^8^ bacteria/100 μL of phosphate buffered saline (PBS). To create mixed gram-negative and gram-positive bacterial infection, 1 × 10^8^ Xen14 bacteria and 1 × 10^8^ Xen29 bacteria/100 μL of PBS were used for wound contamination. Three animal models were used to create bacterial infection routes: subcutaneous injection (hereafter referred to as (I)), cut wound (hereafter referred to as (C)), and skin grafting (hereafter referred to as (S)). In the (I) model, *E. coli* and/or *S. aureus* suspensions were injected subcutaneously into the backs of the mice using an Fr. 25 needle. In the (C) model, a 1 cm incision wound was created in the midline of the back, smeared with *E. coli* and/or *S. aureus* suspension, and the wound was closed directly with a 4–0 nylon suture. In the (S) model, a 1 × 1 cm rectangular full- thickness skin graft was lifted from the backs of the mice, *E. coli* and/or *S. aureus* suspensions were spread over the wound bed, and the skin graft was reattached and closed with a 4–0 nylon suture. An additional group of animals in each of these three models was inoculated with PBS to serve as a negative control. The mice had *ad libitum* access to food and water both before and after the surgery or administration of bacterial infection. The mice were killed 24 h after the administration of bacterial infection, and the whole blood was drawn and collected in RNAprotect Animal Blood Tubes (cat.No. 76544, Qiagen, USA) without anticoagulant. After the whole blood samples were incubated at room temperature for 15 min, they were centrifuged at 3000 × *g* for 10 min, white blood cells were slowly removed from the corresponding layers, and the serum was extracted and stored at −80°C before processing for RNA analyses. All the housing conditions and the surgical procedures, analgesia, and assessments were in accordance with national and institutional guidelines, and an Association for Assessment and Accreditation of Laboratory Animal Care (AAALAC)-accredited SPF facility was used. The animal protocols were approved by the Institutional Animal Care and Use Committee (IACUC) of Kaohsiung Chang Gung Memorial Hospital.

### RNA isolation

Total RNA was extracted from the harvested serum using a mirVana miRNA Isolation Kit (Ambion, USA). The purified RNA yield was determined by the absorbance at 260 nm with an SSP-3000 NanoDrop spectrophotometer (Infinigen Biotech, USA), and RNA quality was evaluated with a BioAnalyzer 2100 system (Agilent Technologies, USA).

### Small RNA library preparation

RNA smaller than 200 base pairs (bp) was enriched with the mirVana miRNA isolation kit (Ambion). The small RNA samples were sent to GeneTech Biotech Co., Ltd (GeneTech, Taiwan) for small RNA cloning. The population of miRNAs with a length of 15–30 nucleotides (nt) was passively eluted from polyacrylamide gels. The RNA was then precipitated with ethanol and dissolved in water. Small RNAs had linkers ligated to them and bar-coded cDNAs were prepared using a TruSeq Small RNA Sample Prep Kit (Illumina, USA) following the manufacturer’s instructions. Briefly, 1 μg of small RNA was ligated with adapters at 3′ and 5′ ends. Adapter-ligated RNA was reverse-transcribed with SuperScript II Reverse Transcriptase (Invitrogen, USA), then PCR-amplified (15 cycles). Samples were barcoded using 15 variants of the reverse primer provided with the kit. The indexing barcode of this kit after adapter ligation could significantly reduce sample bias over previous indexing/barcoding approaches where a barcode was ligated directly to the miRNA [21]. Individual libraries were analyzed on a BioAnalyzer (Agilent) for the presence of linked cDNA at the appropriate size (135–165 bp) and 11 bar-coded libraries were pooled into 1 sample by mixing 2.0 ng of the 135–165 bp peak from each sample, as determined by the BioAnalyzer.

### Illumina small RNA deep sequencing

Sequencing of the pooled libraries was performed in 1 lane of the Illumina HiSeq2000 Sequencer. Fifty bp single-end reads of the libraries were obtained. After indexing and trimming of linker sequences, those reads of at least 15 nt in length that had less than three terminal mismatches in the sequence were sorted and counted for the following analysis. The clean reads were aligned against the reference genome indices for mouse (*M. musculus*, UCSC mm9) provided by the Bowtie site (http://bowtie-bio.sourceforge.net/index.shtml) using BOWTIE software according to the following criteria: a 5′ and 3′ linker match of at least 15 nt and an appropriate length (15–29 nt). The pre-miRNAs and mature miRNAs in the miRBase v.20.0 were searched with BLAST to identify *Mus musculus* miRNAs. To evaluate the quality of deep sequencing experiments, five miRNAs detected by small RNA deep sequencing were randomly selected and quantified by qPCR using the Applied Biosystems 7500 Real-Time PCR System (Life Technologies, USA) to confirm the up-regulation of miRNA expression in Xen29 (I) group vs. Xen29 (C) group. Twenty-five femto-molar of single stranded cel-miR-39 synthesized by Invitrogen (Invitrogen) was spiked into 400 μL of serum as an internal control for the expression of each miRNA.

## Results

### Deep sequence analysis of small RNAs

**Figure 1:**
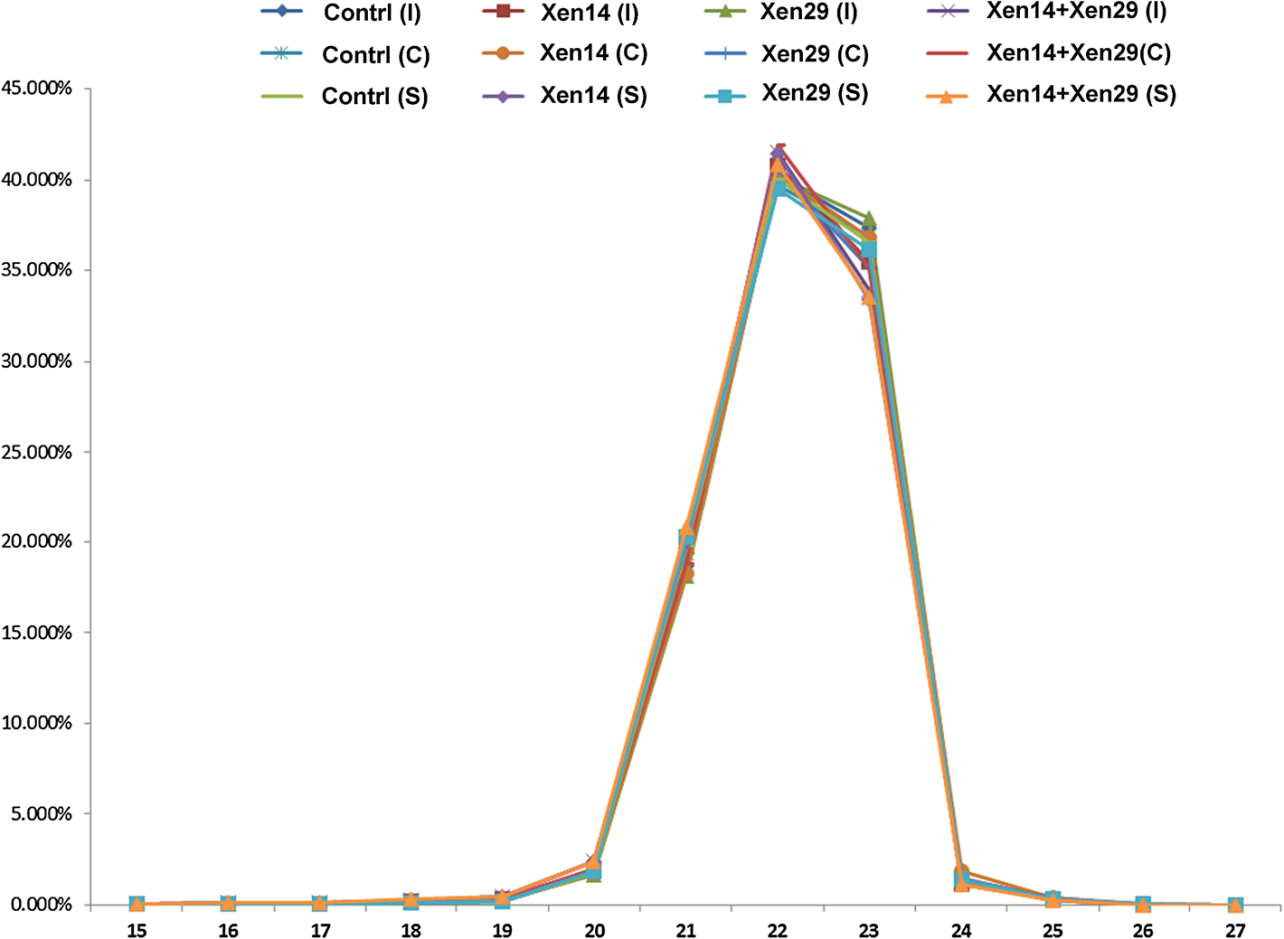
**Length distribution and abundance of small RNA sequences by Illumina small RNA deep sequencing in 12 libraries.** Xen14: recombinant-specific gram-negative pathogen *Escherichia coli*; Xen29: recombinant-specific gram-positive pathogen *Staphylococcus aureus*. Three animal models were used to create bacterial infection routes: subcutaneous injection (I), cut wound (C), and skin graft (S), with 1 × 10^8^ Xen14 and/or Xen29 bacteria in 100 μL of phosphate buffered saline (PBS).

To profile the circulating miRNAs expressed during gram-negative and gram-positive bacterial infection, 12 small RNA libraries were generated from the serum of mice after exposure to PBS, Xen14, Xen29, and Xen14 + Xen29 via the routes of subcutaneous injection (I), cut wound (C), or under grafted skin (S). These 12 small RNA libraries were indicated as Contrl (I), Xen14 (I), Xen29 (I), Xen14 + Xen29 (I), Contrl (C), Xen14 (C), Xen29 (C), Xen14 + Xen29 (C), Contrl (S), Xen14 (S), Xen29 (S), and Xen14 + Xen29 (S). The number and proportions of the categories of small RNAs found are given in the Additional file 1. In total, around 5424663 to 5999008 high-quality raw reads were obtained from the serum libraries. After filtering the low-quality sequences, empty adaptors and single-read sequences, 92.58% to 97.85% clean reads of 15–29 nt were selected for further analysis (Additional file 1: Table S1). The selected reads from these serum libraries mapped well to the mice genome, amounting to 74.19% and 79.32% of the total reads. The rest of the sequences were found to be other types of RNA, including noncoding RNA, rRNA, scRNA, snRNA, snoRNA, srpRNA, and tRNA. The size distribution of small RNAs (sRNAs) was similar in the 2 libraries, and the majority of them were from 21 to 23 nt (Figure 1). The most abundant size class was 22 nt, which accounted for ~40% of the total reads in these libraries, followed by 23 nt for ~35% (Additional file 1: Table S2). This result is typical of Dicer-processed small RNA products and was consistent with the known ~22 nt for miRNAs.

### Identification of known miRNAs

The Illumina small RNA deep sequencing approach allows us to determine the relative abundance of various miRNA families by calculating the sequencing frequency. To investigate the expression of known miRNAs during bacterial infection, identified small RNA sequences were compared with known mature miRNAs in miRBase v.20.0. The measured 814 mature miRNAs from these 12 libraries are listed (Additional file 1: Table S3). Among them, there were 52 mature miRNAs with sequence reads ≥ 400 (Additional file 1: Table S4) and 10 mature miRNAs (mir-10b-5p, mir-133a-1-3p, mir-133a-2-3p, mir-191-5p, mir-22-3p, mir-25-3p, mir-3107-5p, mir-486-5p, mir-92a-1-3p, mir-92a-2-3p) with sequence reads ≧ 4000 in at least 1 of the 12 twelve libraries (Additional file 1: Table S5). In these libraries, known miRNAs had a broad range of expression level; some (such as mir-486-5p and mir-3107-5p) were found to have hundreds of thousands of sequence reads, while many others had less than 20, indicating that expression varies significantly among different miRNA families. With more than 6000 reads, 3 miRNAs (mir-486-5p, mir-3107-5p, and mir-92a-3p) were dominantly expressed in all these 12 libraries (Additional file 1: Table S6). In addition to mir-486-5p and mir-3107-5p, which have more than 200000 sequence reads in all 12 libraries, mir-92a-3p was the third most abundant miRNA with sequence reads ranging from 6659 to 16734 (Additional file 1: Table S6). Small RNA deep sequencing and qPCR results of five selected miRNAs (mir-133a-1-3p, mir-127-3p, mir-25-3p, mir-191-5p, and mir-215-5p) were generally in agreement, with a Pearson correlation value of 0.921 (Additional file 2).

### Differentially-expressed miRNAs after bacterial infection

**Figure 2:**
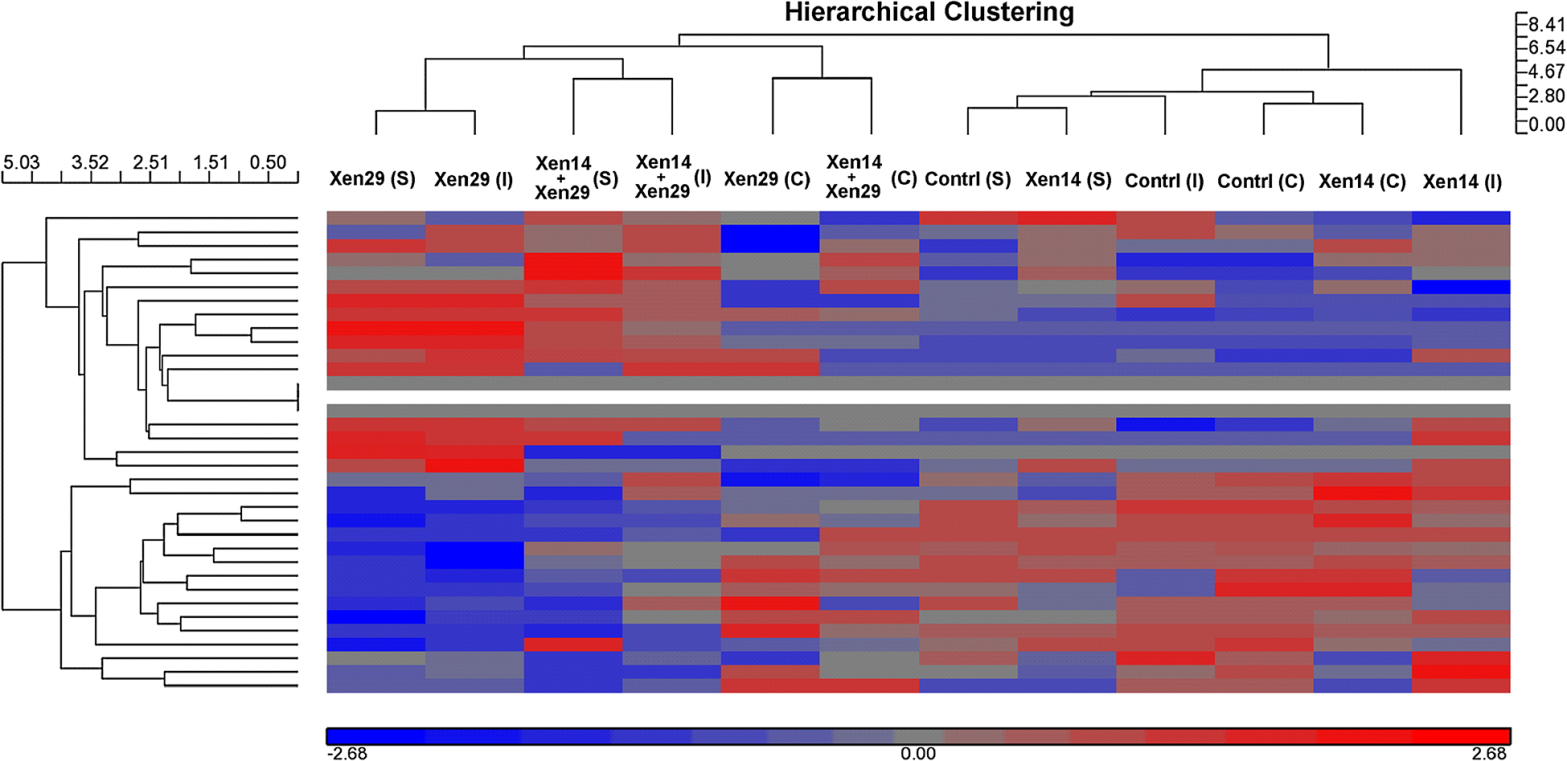
**Unsupervised hierarchical clustering of the expression of miRNAs.** Hierarchical clustering of miRNA differentially expressed in the sera of the mice receiving Xen14, Xen29, Xen14 + Xen29, or PBS (as control) inoculation via subcutaneous injection (I), cut wound (C), or skin graft (S).

**Figure 3:**
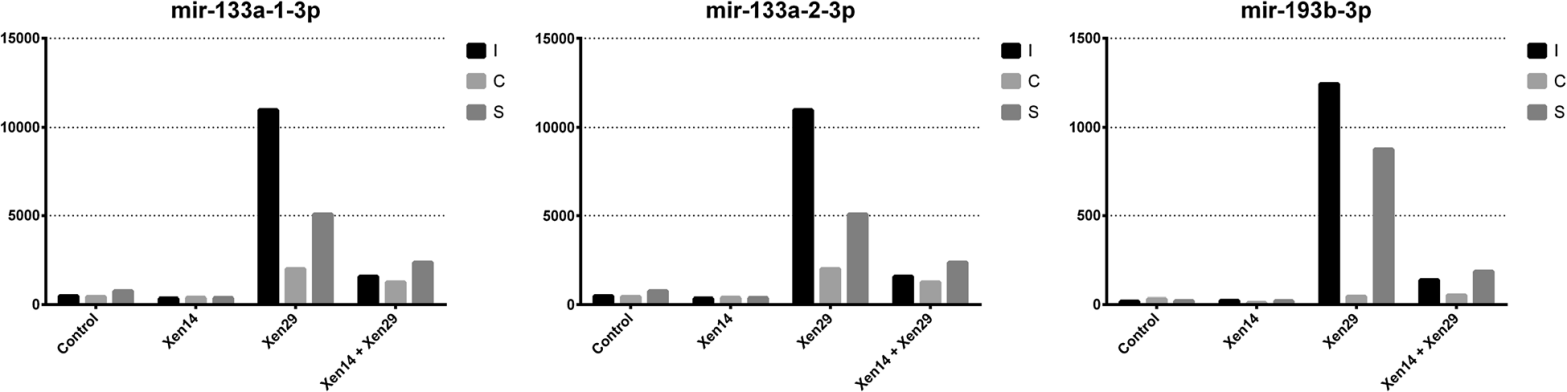
**Comparison of the sequence reads as the expression levels of 3 dominant circulation miRNAs (mir-133a-1-3p, mir-133a-2-3p, and mir-193b-3p) in the sera of the mice receiving bacterial infection.**

According to the changes in relative miRNA abundance between the serum libraries from the mice receiving bacterial infection (Xen14, Xen29, and Xen14 + Xen29) and PBS injection after 24 h, the differentially-expressed miRNAs with sequence reads more than 400 in at least 1 of the libraries were selected for further comparison. It was revealed that a total of 9 miRNAs (mir-193b-3p, mir-133a-1-3p, mir-133a-2-3p, mir-133a-1-5p, mir-133b-3p, mir-434-3p, mir-127-3p, mir-676-3p, mir-215-5p) showed differences greater than 4-fold with *p*-value < 0.01 between the 2 libraries (Table 1). Following exposure to gram-negative bacteria alone, no differentially-expressed miRNAs were found in the injection, cut, or skin graft models. Following exposure to gram-positive bacteria in the injection and skin graft models, 7 upregulated miRNAs (mir-193b-3p, mir-133a-1-3p, mir-133a-2-3p, mir-133b-3p, mir-434-3p, mir-127-3p, mir-676-3p) and 1 downregulated miRNA (mir-215-5p) were found. In addition, following exposure to gram-positive bacteria in the cut model, only 2 miRNAs (mir-133a-1-3p, mir-133a-2-3p) were upregulated and 1 miRNA (mir-215-5p) was downregulated. Exposure to mixed bacteria induced a similar expression pattern of the circulating miRNAs to that induced by gram-positive bacterial infection, but not to that induced by gram-negative bacterial infection. Unsupervised hierarchical cluster analysis (Figure 2) of all significant differentially-expressed miRNAs also revealed the mixed gram-negative and gram-positive bacteria induced a similar expression pattern of the circulating miRNAs to that induced by gram-positive bacterial infection, and gram-negative bacteria induced a similar expression pattern of the circulating miRNAs to that induced by the PBS in the control. Among these miRNAs, mir-193b-3p had the highest fold-change of 61.5-fold and 40.4-fold in the injection and skin graft models of gram-positive bacterial infection, respectively, and of 13.9-fold and 13.0-fold in the injection and skin graft models of mixed bacterial infection, followed by mir-133a-1-3p and mir-133a-2-3p in the gram-positive or mixed bacterial infection. Therefore, we focused on these 3 differentially-expressed miRNAs (mir-193b-3p, mir-133a-1-3p, mir-133a-2-3p), and their expression with sequence reads is illustrated in Figure 3. Obviously, the expression of mir-133a-1-3p and mir-133a-2-3p is similar in the 12 libraries. In addition, the high expression of mir-133a-1-3p and mir-133a-2-3p upon gram-positive bacterial infection is reduced, although still significant, in those mice with mixed bacterial infection.

**Table 1:** **Differentially expressed miRNAs with sequence read > 400 and regulated greater than 4-fold in the sera of C57BL/6 mice receiving bacterial infection for 24 h**

**Xen14**: **(I)** Injection subcutaneously			**(C)** Cut			**(G)** Grafting of skin		
miR-name	Fold-change	*p*	miR-name	Fold-change	*p*	miR-name	Fold-change	*p*
None			None			None		
**Xen29**: **(I)** Injection subcutaneously			**(C)** Cut			**(G)** Grafting of skin		
miR-name	Fold-change	*p*	miR-name	Fold-change	*p*	miR-name	Fold-change	*p*
mmu-mir-193b-3p	61.5	**	mmu-mir-133a-2-3p	4.7	**	mmu-mir-193b-3p	40.4	**
mmu-mir-133a-2-3p	21.0	**	mmu-mir-133a-1-3p	4.7	**	mmu-mir-133b-3p	7.0	**
mmu-mir-133a-1-3p	21.0	**	mmu-mir-215-5p	0.1	**	mmu-mir-133a-2-3p	6.6	**
mmu-mir-133b-3p	8.9	**				mmu-mir-133a-1-3p	6.6	**
mmu-mir-434-3p	6.2	**				mmu-mir-434-3p	5.7	**
mmu-mir-127-3p	5.5	**				mmu-mir-676-3p	5.2	**
mmu-mir-676-3p	5.0	**				mmu-mir-127-3p	5.1	**
mmu-mir-215-5p	0.1	**				mmu-mir-215-5p	0.1	**
**Xen14 + Xen29**: **(I)** Injection subcutaneously			**(C)** Cut			**(G)** Grafting of skin		
miR-name	Fold-change	*p*	miR-name	Fold-change	*p*	miR-name	Fold-change	*p*
mmu-mir-193b-3p	13.9	**	mmu-mir-133a-2-3p	4.2	**	mmu-mir-193b-3p	13.0	**
mmu-mir-133a-2-3p	6.1	**	mmu-mir-133a-1-3p	4.2	**	mmu-mir-133a-1-5p	9.6	**
mmu-mir-133a-1-3p	6.1	**	mmu-mir-215-5p	0.2	**	mmu-mir-133a-2-3p	4.7	**
mmu-mir-215-5p	0.1	**				mmu-mir-133a-1-3p	4.7	**
						mmu-mir-133b-3p	4.2	**
						mmu-mir-434-3p	4.0	**

**, *p*-value < 0.01.

## Discussion

The analysis of the serum libraries from the mice receiving bacterial infection (Xen14, Xen29, and Xen14 + Xen29) against those with PBS injection by small RNA deep sequencing after 24 h identified differentially-expressed miRNAs. Following exposure to gram-negative bacteria alone, no differentially-expressed miRNAs were found in the injection, cut, or skin graft models. This unexpected result from gram-negative bacterial infection may be partly attributed to characteristics of the selected skin infection models (subcutaneous injection, cut, and skin graft), which were generally responsive to inoculation by the gram-positive bacteria, but not the gram-negative bacteria. Investigation of the up-regulated circulating miRNAs with different kinds of animal model which facilitate a gram-negative bacteria inoculation, such as peritoneal injection, enterocolitis, or induced pneumonia may be useful to clarify the difference. In addition, the results seemed to contrast to our previous report that LPS injection induced up-regulation of the miRNAs (let-7d, miR-15b, miR-16, miR-25, miR-92a, miR-103, miR-107 and miR-451) of the whole blood in a dose- and time-dependent manner [19]. In a single LPS injection, circulating miRNA induction occurred after 2 h, persisted for at least 6 h, and declined 24 h [19]. Considering the amount of bacteria may vary under the competition of bacterial growth and host defense after inoculation, further experiment with *in vivo* monitor at different observation time points may be helpful to give more information. In addition, for the same reasons, it was expected and observed in this study that the exposure to mixed bacteria induced a similar expression pattern of the circulating miRNAs to that induced by gram-positive bacterial infection. However, it is noted that the number of upregulated miRNAs and their high level of expression upon gram-positive bacterial infection was remarkably decreased in those mice with mixed bacterial infection, suggesting that the competition of different types of bacteria has an impact on the expression of circulation miRNA.

In this study, there were 8 upregulated miRNAs (mir-193b-3p, mir-133a-1-3p, mir-133a-2-3p, mir-133a-1-5p, mir-133b-3p, mir-434-3p, mir-127-3p, mir-676-3p) and 1 downregulated miRNA (mir-215-5p) present as potential targets for differentiation between gram-negative and gram-positive bacterial infection. The analysis of multiple miRNAs in parallel to increase sensitivity and specificity by using complex miRNA expression patterns might constitute very useful and accessible diagnostic tools in a cluster pattern [5,7]. However, the detection of the type of infected bacteria should not be interfered from the animal model approach, particularly since the inoculation methods such as subcutaneous injection, cut, or skin grafting were not expected to have a great impact on circulating miRNA expression. Therefore, the 3 most common circulating miRNAs (mir-193b-3p, mir-133a-1-3p, and mir-133a-2-3p) expressed in the mice exposed to *Staphylococcus aureus* in the absence or presence of *Escherichia coli* may be potential biomarkers for gram-positive bacterial infection. However, whether these 3 circulating miRNAs would expressed in the mice exposed to other species of gram-positive bacteria require further validation.

A functional role of miR-133a and miR-193b was recently revealed in systemic inflammatory responses associated with infections, myocardial infarction, and cancer. Significant alterations of miR-133a, miR-193b, miR-150, and miR-155 were found in mice after cecal pole ligation and puncture-induced sepsis [12]. Although miR-193b has been considered a tumor suppressor gene, and modulates proliferation, migration, and invasion of the cancer cells [22-25], it has been reported to be associated with death from sepsis in an analysis of 214 sepsis patients (117 survivors and 97 non-survivors based on 28-day mortality) [26]. Furthermore, miR-133a is deemed a cardiac and skeletal muscle-specific miRNA [27,28] and involved in muscle development [29] and many myocardial diseases [29-32]. Significantly elevated miR-133a levels were found in critically ill patients at intensive care unit (ICU) admission, especially in patients with sepsis [12]. In addition, correlation analyses revealed significant correlations of miR-133a with disease severity, classical markers of inflammation and bacterial infection, and organ failure [12]. Notably, genes encoding miR-133, namely *miR-133a-1*, *miR-133a-2*, are transcribed as bicistronic transcripts together with miR-1-1 and miR-1-2 [29]. The remarked increase in expression of mir-133a-1-3p and mir-133a-2-3p upon gram-positive bacterial infection in the present study was not accompanied by an increased expression of mir-1-1 or mir-1-2. Whether the upregulation of these circulating miRNAs is attributable to direct stimulation by gram-positive bacterial toxin or by a parallel effect, such as hemodynamic change or an associated illness, is unknown. Therefore, the origin and mechanism of the increased expression of mir-133a-1-3p and mir-133a-2-3p may require further investigation prior to their application in a clinical setting.

## Conclusion

In conclusion, this study profiled circulating miRNAs in mice exposed to gram-negative and gram-positive bacteria using Illumina small RNA deep sequencing, and identified the miRNAs that may potentially be used as biomarkers of gram-positive bacterial infections.

## Additional files

Additional file 1: Table S1. Summary of Illumina small RNA deep sequencing data for small RNAs in the sera of C57BL/6 mice receiving Xen14 or/and Xen29 bacteria infection by subcutaneous injection, cut, or skin grafting. **Table S2.** The distribution of the sequence lengths within 15–29 nt read by the RNA deep sequencing in the 12 libraries of sera of C57BL/6 mice receiving Xen14 or/and Xen29 bacteria infection by subcutaneous injection, cut, or skin grafting. **Table S3.** The expression patterns of known miRNAs in the 12 libraries of sera of C57BL/6 mice receiving Xen14 or/and Xen29 bacteria infection by subcutaneous injection, cut, or skin grafting. **Table S4.** The expression patterns of known miRNAs with sequence reads > 400 in at least one of the 12 libraries of sera of C57BL/6 mice receiving Xen14 or/and Xen29 bacteria infection by subcutaneous injection, cut, or skin grafting. **Table S5.** The expression patterns of known miRNAs with sequence reads > 4000 in at least one of the 12 libraries of sera of C57BL/6 mice receiving Xen14 or/and Xen29 bacteria infection by subcutaneous injection, cut, or skin grafting. **Table S6.** The most top 15 expressed miRNAs with sequence reads in each of the 12 libraries of sera of C57BL/6 mice receiving Xen14 or/and Xen29 bacteria infection by subcutaneous injection, cut, or skin grafting. Additional file 2:
**Correlation of the expression of five selected miRNAs in small RNA deep sequencing and qPCR.**
