# Supplementary figures and images for: Profiling the circulating miRNAs in mice exposed to gram-positive and gram-negative bacteria by Illumina small RNA deep sequencing
Source: J Biomed Sci. 2015 Jan 7;22(1):1. doi: 10.1186/s12929-014-0106-y (PMC4300083; doi:10.1186/s12929-014-0106-y)

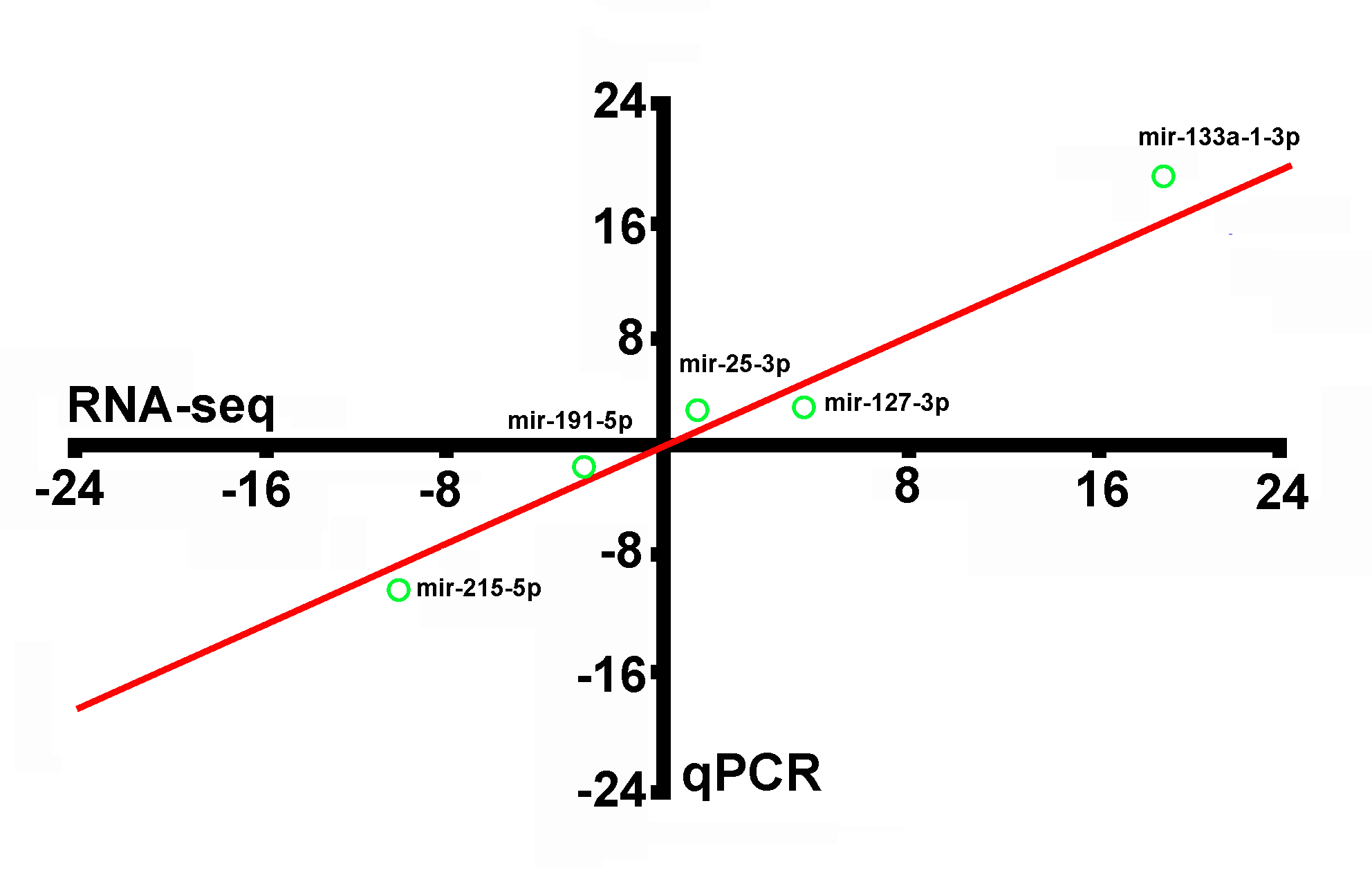

Supplement: Additional file 2: — Correlation of the expression of five selected miRNAs in small RNA deep sequencing and qPCR. [file 12929_2014_106_MOESM2_ESM.tiff]
